# Effectiveness of the InCharge Prevention Program to Promote Healthier Lifestyles: Protocol for a Randomized Controlled Trial

**DOI:** 10.2196/17702

**Published:** 2020-07-08

**Authors:** Mathijs Mesman, Simone Onrust, Renée Verkerk, Hanneke Hendriks, Bas Van den Putte

**Affiliations:** 1 Amsterdam School of Communication Research University of Amsterdam Amsterdam Netherlands; 2 Trimbos Institute Utrecht Netherlands

**Keywords:** school-based health intervention, adolescents, health behavior, healthy lifestyle, quality of life, behavior change

## Abstract

**Background:**

InCharge is a newly developed school-based health intervention aimed at older adolescents. It aims to promote a healthier lifestyle by increasing self-regulation skills. After the InCharge program’s effectiveness was previously investigated in a pilot study, the content of the program was adapted.

**Objective:**

This study describes the protocol of a cluster randomized controlled trial that aims to investigate the effectiveness of the InCharge program.

**Methods:**

A cluster randomized controlled trial including 70 classes with older adolescents (aged 16 years or older) in the Netherlands will be conducted to test the effectiveness of the InCharge program. After schools are recruited, randomization occurs at the class level. The trial consists of the following two conditions: an experimental condition and a control condition. Participants in the experimental condition will be given the InCharge intervention, consisting of four lessons of 50 minutes, with each lesson containing three assignments of approximately 15 minutes. While participants in the experimental condition will receive InCharge, participants in the control condition will receive regular academic school courses. Surveys are administered 1 week before the intervention (baseline), 1 week after the intervention (posttest), and 12 weeks after the intervention (follow-up). Variables of interest include, but are not limited to, self-regulation; predictors of snack intake, physical activity, and alcohol use; and interpersonal communication regarding these health behaviors. In addition to surveys, observations will be conducted during the first and fourth lessons, teachers will be interviewed, and focus groups will be held with a selection of students from the intervention condition.

**Results:**

Enrollment started in September 2017. As of June 2019, a total of 1216 participants were enrolled for this trial. Findings will be published in peer-reviewed journals and presented at conferences. The trial has been approved by the Ethics Review Board of the Faculty of Social and Behavioral Sciences of the University of Amsterdam (reference no.: 2017-PC-8244).

**Conclusions:**

In this study protocol, the design of a cluster randomized controlled trial is described, which assesses how effectively the school-based intervention InCharge stimulates healthier lifestyles in late adolescents. We hypothesize that participants in the experimental condition will consume less alcohol, eat fewer unhealthy snacks, and be more physically active compared with participants in the control condition.

**Trial Registration:**

Netherlands Trial Register (NL6654); https://www.trialregister.nl/trial/6654

**International Registered Report Identifier (IRRID):**

RR1-10.2196/17702

## Introduction

### Background

Health and mortality are strongly affected by behaviors such as excessive alcohol use, a poor diet, and physical inactivity [1]. Some health-risk behaviors, such as a poor diet, originate early in life and continue to deteriorate as children grow older [2]. Most health-risk behaviors, however, develop in adolescence. When children move into adolescence, they become less dependent on their parents, peer relationships gain importance, and exposure to high-risk behaviors increases [3]. Generally, unhealthy lifestyle behaviors steadily increase through adolescence, with a peak in late adolescence (age 16 years or older) [4,5]. During this developmental period, several changes in adolescents’ social environment contribute to the accumulation of unhealthy lifestyle behaviors. For example, in late adolescence, most individuals transition from secondary schooling to further education, and the number of adolescents with a part-time job increases with age. Furthermore, older adolescents spend less time in their family home and are increasingly allowed to make their own choices. At the same time, more social activities occur in drinking contexts, and together these changes are associated with unhealthy lifestyle behaviors, such as an escalation of alcohol use [6], a poor diet [7], and decreases in physical activity [8].

### Effectiveness of Health Promotion Programs

In the past decades, numerous health promotion programs have been developed in order to prevent the rapid increase of unhealthy behaviors during adolescence. The majority of these programs take place in the school environment, because many adolescents can easily be reached by school-based health interventions. Several studies have suggested that these interventions can indeed promote healthier lifestyles, such as a healthy diet [9] and physical activity [10], and prevent health-risk behaviors, such as alcohol use [11]. However, most systematic reviews and meta-analyses demonstrated large heterogeneity in effectiveness [10,12].

Furthermore, most health promotion programs include several different components, and in most evaluation studies, the exact relationship between these intervention components and their effects remains unclear. In order to understand which program components contribute to healthier lifestyle behaviors and which components do not, several authors conducted meta-regression analyses. For example, previous research demonstrated that self-regulation, which is defined as the capacity needed to resist temptations and impulses [13], was an effective component in health interventions [14]. Among all prevention strategies that were included in the meta-regression, self-monitoring (ie, observing and recording a target behavior [15]) contributed the most to program effectiveness, and combining self-monitoring with at least one other technique based on self-regulation resulted in the largest effect sizes for both healthy eating and physical activity. Furthermore, research showed that health programs based on improving self-regulation skills were especially effective in late adolescence [12].

Therefore, the school-based health intervention investigated in this protocol study also incorporated self-regulation as the main prevention strategy to promote healthier lifestyles. The intervention, called “InCharge,” is newly developed by the Trimbos Institute (the Netherlands Institute for Mental Health and Addiction). The InCharge program is developed for older adolescents and aims to promote healthier lifestyles by improving self-regulation skills (ie, through elaborating on and practicing self-regulation). The program helps students realize what is important to them and how certain temptations can hinder them from achieving these goals, teaches them that self-regulation can be used to resist certain temptations (eg, peer pressure), trains self-regulation skills by formulating an action plan, and helps them practice self-regulation through various assignments. Finally, alcohol is introduced as a temptation, and students formulate action plans for responsible alcohol use in order to stimulate healthier drinking behaviors.

This protocol study also investigates potential factors that determine the effectiveness of the InCharge school-based health intervention, because previous studies have found large heterogeneity in the effectiveness of these types of interventions [12]. In particular, this protocol study investigates the role of interpersonal communication during the InCharge school-based health intervention. In the context of mass-mediated health interventions, previous studies have shown that interpersonally communicating about health-related topics is strongly related to health behavior [16,17] and that health interventions can indirectly influence health behaviors through stimulating interpersonal communication about a health behavior [18]. This protocol study will investigate similar purposes of interpersonal communication in a school-based health context. Furthermore, as most school-based health interventions are taught by school teachers, this study protocol will also investigate the role of teacher-related communication during the InCharge program.

### Theoretical Basis

The InCharge program is based on several psychological theories such as the social cognitive theory (SCT) [19], the theory of planned behavior (TPB) [20], and the goal-setting theory [21]. The SCT explains behavior by the interaction of personal factors, such as outcome expectancies, self-efficacy, and environmental factors, such as the behavior of others. According to the SCT, behavior change can occur through both active learning and modeling (ie, vicarious reinforcement by learning from the experiences of others). Both mechanisms are incorporated in InCharge; the 7-day challenge offers the opportunity to actively learn through practicing self-control, discussing experiences of classmates with the challenge, and watching several video fragments, resulting in vicarious reinforcement. The TPB is commonly used to explain health behavior and postulates that intention, the most important determinant of behavior, is in turn influenced by the following three constructs: attitude, subjective norms, and perceived behavioral control, which is comparable to self-efficacy in SCT. InCharge mainly aims to influence perceived behavioral control by elaborating on and practicing self-regulation skills. As research has shown that outcome expectancies are related to attitudes [22], the program is expected to influence attitudes by linking the consequences of giving in to health-related temptations to personal goals. Subjective norms are influenced through discussions about social acceptability (regarding alcohol use). Finally, the goal-setting theory explains behavior change by means of an action sequence of wishing, planning, acting, and evaluating [23]. Goals should be both challenging and feasible [24], and plans should connect a certain goal-directed activity with an anticipated situation [25] in order to be successful. These principles are incorporated in the 7-day challenge, which is one of the components of InCharge.

The InCharge program is developed by taking into account the developmental characteristics of older adolescents. The primary developmental tasks of late adolescence are forming an identity, planning the future, and acquiring the necessary skills to transition into adulthood [26]. As older adolescents experience fundamental changes in their self-definition and identity by exploring new philosophies, lifestyles, and behaviors [6], late adolescence is ideally suited to introduce a healthy lifestyle. Previous research on substance use prevention demonstrated that older adolescents benefit from a social influence approach [12], which makes them aware of the various social pressures to use substances in order to be psychologically prepared to resist these influences. The InCharge program also creates awareness of peer pressure by showing and discussing different examples of peer pressure. In late adolescence, the brain considerably matures, improving executive functions, such as planning, thinking ahead, response inhibition, and more advanced self-regulation and impulse control [27,28]. As a result, older adolescents are capable of mastering self-regulation skills, which has been proven to be an effective prevention strategy in this developmental period [12]. Therefore, improving students’ self-regulation skills is one of the core prevention strategies of InCharge.

### Aim and Hypotheses

This study describes the protocol of a randomized controlled trial (RCT) including 70 classes with older adolescents, which aims to evaluate the effectiveness of the InCharge program. Baseline, posttest, and 3-month follow-up measures will be conducted to determine whether the program successfully influences the determinants of health behaviors. After completing the program, students in the intervention condition are expected to refrain more from binge drinking and eating unhealthy foods, such as snacks and sweets, and to be more physically active than students in the control condition.

Other aims of our study are to understand the mechanisms through which the InCharge program has its effects. We investigate whether the effects of the program on health behaviors can be explained by increased self-regulation, which is expected to be one of the core mechanisms of the program. Additionally, as previous research has shown the importance of interpersonal communication in mass-mediated health interventions [18], our study aims to investigate interpersonal communication in the context of a school-based health intervention.

## Methods

### Study Design

**Figure 1:**
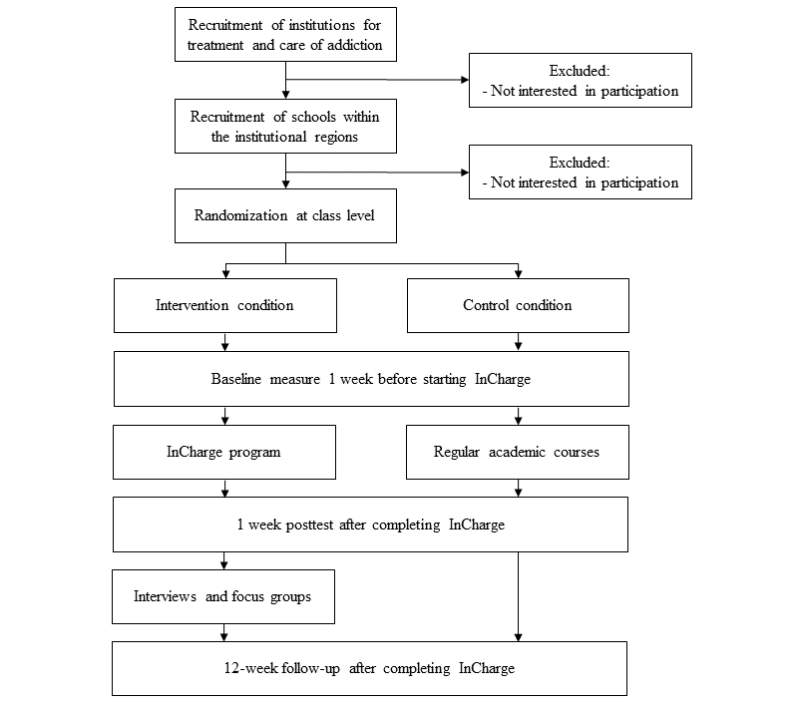
Design of the cluster randomized controlled trial.

The InCharge effectiveness study is a cluster RCT including the following two conditions: an experimental condition (the InCharge program) and a control condition (no intervention) (Figure 1). Participants are older adolescents (aged 16 years or older) in schools for intermediate vocational education, schools for higher general secondary education, and schools for preuniversity education. After initial recruitment and enrollment of schools in the trial, randomization takes place at the class level. In each school, measurements in both experimental and control classes are scheduled 1 week before the four lessons of the InCharge program are implemented in the experimental classes (baseline), and 1 week (posttest) and 3 months (follow-up) after the experimental classes have completed the InCharge program. In addition, the first and fourth lessons of the program are observed by two independent coders in order to obtain information on how the program is delivered, interviews are conducted with teachers about their experiences with the program, and focus groups are conducted with small groups of students about their experiences with the program (both interviews and focus groups are conducted 1 week after the posttest assessment). After all data have been collected within a school, control classes will also receive the intervention.

### Participants

#### Recruitment

The Municipal Health Services and prevention departments of regional institutions for the treatment and care of addiction help to implement the InCharge program in schools. From these Municipal Health Services and regional institutions, 47 prevention practitioners are trained by the Trimbos Institute to instruct teachers on how to deliver the program and are asked to provide us with a list of schools in their region interested in participating in the InCharge study. The research team contacts these schools to explain the objective and design of the study. First, schools receive an invitation and an information brochure via email, and thereafter, schools are contacted by phone to discuss participation in the study. Schools are allowed to select the number of classes participating in the study, with a minimum of two classes because randomization occurs within schools. To create comparable research conditions, schools are instructed that a minimum number of two classes should be from the same educational level of the field of study. Classes are eligible for inclusion in the study if they contain older adolescents and the level of education is intermediate vocational education, higher general secondary education, or preuniversity education. Students are recruited by class participation. In collaboration with the schools, students’ parents are informed about the study objectives and receive passive informed consent forms. Written active informed consent is obtained from all participating students.

#### Randomization

We randomize the participants at the class level, and classes within schools are matched on the school level in order to obtain more comparable research conditions. Classes are randomly allocated to the experimental or control condition by means of repeated coin tossing. For each selection of matched classes within schools, classes are assigned to the experimental condition if the coin toss shows heads and to the control condition if the coin toss shows tails. The coin toss procedure is continued until all matched classes are assigned to a research condition, such that as many matched classes as possible are assigned to the experimental and control conditions. Randomization is carried out centrally, with two researchers present to monitor the procedure.

### Sample Size Calculation

Effect sizes are difficult to estimate for the InCharge program because the program is newly developed. As school-based prevention programs generally have small effects [12], we based the estimation of the sample size on a small effect size (*d*=0.2) [29]. In order to reliably detect a small-sized effect in a two-sided test with a conventional significance threshold of *α*=.05 and a power of (1–*β*)=0.80, 393 students are required per condition. However, as students are nested in classes and the program is delivered to classes of students, observations of students in the same class are not independent. Therefore, the required sample size is corrected for clustering. Assuming an intraclass correlation of 0.025 and an average class size of 16 participating students, the required sample size increases to 560 students in 35 classes per condition. Calculations are made using the downloadable procedure “clustersampsi” from Stata (StataCorp).

### Study Intervention

The experimental intervention is a school-based program named InCharge, which has been designed for older adolescents. The main objective of the program is to stimulate healthy lifestyle behaviors, such as increased physical activity, and to decrease unhealthy behaviors, such as binge drinking and eating unhealthy foods. These objectives should mainly be obtained by means of increasing students’ self-regulation skills.

### InCharge Program

The InCharge program consists of four 50-minute lessons, and each lesson contains three assignments of approximately 15 minutes. Teachers are trained on how to work with the program material by prevention professionals from Municipal Health Services or regional institutions for the treatment and care of addiction. Materials, such as worksheets, videos, and a web-based quiz tool, are available on a digital teacher platform. In addition, a specially designed app for the smartphone (the 7-day challenge) is used to help students complete the program.

In the first assignment of the first lesson, students visualize their goals for the next 5 years by thinking about what they want to achieve in life and why this is important to them. In the second assignment, students discuss the biggest temptations in their lives. For this assignment, the teacher clears a space in the classroom and draws an imaginary line. Students are asked to position themselves alongside this imaginary line according to how tempting they assess certain temptations, such as chocolate or sweets, alcohol, and staying in bed in the morning. After each temptation, the teacher asks the students to explain their position. In the third assignment, students visualize how their goals would be affected by repeatedly giving in to their biggest temptations or repeatedly resisting their biggest temptations.

In the second lesson, the concept of self-regulation is introduced (for students, self-regulation is called willpower). First, students watch and discuss a video of the marshmallow experiment to understand the role of self-regulation [30]. In this experiment, young children are offered a choice between one marshmallow provided immediately or two marshmallows after waiting for a short period of time. The second assignment focuses on previous experiences with willpower. Students are asked to discuss their own experiences with succeeded challenges, together with the applied strategies that helped them succeed. Finally, all students prepare a challenge to complete during the next week to train their self-regulation abilities. First, they set a realistic goal for themselves related to a chosen health behavior, after which they create a specific plan to achieve this goal. This plan includes the identification of difficult situations and the selection of coping strategies to address these situations accordingly. The actual challenge is completed as a homework assignment. With the help of a specially designed smartphone app (the 7-day challenge), students monitor their progress.

In the third lesson, first, the results of the 7-day challenge are discussed. During the second assignment, students reflect on their original plan to complete the challenge and discuss how these plans could further be improved. The third assignment addresses peer pressure, as peers have been shown to strongly influence the health behaviors of adolescents [31,32]. In order to grasp the influence of peer pressure, students watch and discuss a video of an experiment with a test subject sitting in a room with confederates. As the room fills up with smoke, all confederates remain calm as if nothing happens. As a result, the test subject also remains seated, which could actually be life threatening in case of a real fire.

The final lesson elaborates on alcohol use. At first, students watch a video of partying adolescents who are consuming large amounts of alcohol. The video shows a group of friends who are having fun at first, but the situation ends with a conflict and an injury as a result of excessive alcohol use. Thereafter, students discuss the role of peer pressure in excessive drinking. Second, in order to make them aware of current social norms, students discuss the social acceptability of binge drinking by means of an interactive web-based quiz. Finally, the students identify situations in their own life, where it is important to be careful with alcohol, and come up with strategies to do so. A more thorough description of the assignments can be found in Multimedia Appendix 1.

### Data Collection

An overview of all measurements is provided in Table 1. Data are collected by means of paper questionnaires, which are answered during school hours under the supervision of a teacher. In addition, observational data are collected during the first and fourth lessons, complemented with qualitative data from individual interviews with teachers and focus groups with a selection of students.

**Table 1:** Overview of measurements.

Measurement		Baseline	Posttest	Follow-up
Demographic variables		X^a^	X	X
**Self-control variables**				
	Perceived self-control/impulsivity	X	X	X
	Perceived temptations	X	X	X
	Motivation to resist temptations	X	X	X
**Alcohol, snacking, and physical activity**				
	Actual behavior	X	X	X
	Intentions	X	X	X
	Attitudes	X	X	X
	Social norms	X	X	X
	Negative outcome expectancies	X	X	X
	Self-efficacy	X	X	X
	Perceived parental rules	X	—^b^	—
**Communication about the program**				
	Frequency (with friends, classmates, and parents)	—	X	—
	Valence (with friends, classmates, and parents)	—	X	—
**Communication about alcohol, snacking, and physical activity**				
	Frequency (with friends, classmates, and parents)	X	X	X
	Valence (with friends, classmates, and parents)	X	X	X
**Program evaluation**				
	Teacher evaluation (student questionnaire)	—	X	—
**Intervention evaluation**				
	Student questionnaire	—	X	—
	Student focus group	—	X	—
	Teacher interview	—	X	—

^a^Measurement performed.

^b^Measurement not performed.

### Outcomes

#### Questionnaire Data

The InCharge program aims to stimulate a healthier lifestyle in general among older adolescents by increasing self-regulation skills. In terms of outcome measures, the study focuses on the following three outcome behaviors: alcohol use, snack intake, and lack of physical activity. A pilot study of the program has shown that these behaviors are most prevalent among older adolescents. The main outcomes that are measured for alcohol use, snack intake, and physical activity are the actual behaviors and predictors of the behaviors. Snack behavior and physical activity are operationalized as the number of times adolescents consume snacks and are physically active in an ordinary week as well as in the past 7 days [33]. Alcohol use is operationalized as the number of times adolescents drink alcohol and engage in binge drinking in the past 4 weeks (ie, frequency), as well as the number of drinks adolescents usually consume on one occasion (ie, quantity). Moreover, the predictors of these behaviors are operationalized according to the TPB as attitudes, subjective norms, perceived behavioral control, and behavioral intention [20]. As an additional predictor, negative outcome expectancies are defined as the anticipated negative result of a certain behavior [34]. We operationalized negative outcome expectancies as the probability that exercising less than once a week or daily snacking will lead to weight increase, a poor condition, and health-related problems. For alcohol use, negative outcome expectancies are operationalized as the probability that drinking more than five glasses of alcohol on one occasion will lead to sickness, loss of control, blackouts, regret, and violence (including sexual violence). Finally, parental rules for the three behaviors are assessed.

Additional measures are general self-control, assessments of various temptations, interpersonal communication about the three health behaviors and the intervention, and evaluation of the program and teacher. First, we assess self-control using an adapted version of the Brief Self-Control Scale [35], which assesses deliberative action and impulse control using the following items: “I am good at resisting temptations,” “I have a hard time breaking bad habits,” “I do certain things that are bad for me, if they are fun,” “I wish I had more self-discipline,” “People would say that I have iron self-discipline,” “Pleasure and fun sometimes keep me from getting work done,” “Sometimes I can’t stop myself from doing something, even if I know it is wrong,” and “I often act without thinking through all the alternatives.” Second, we assess the perceived strength of the temptations with respect to alcohol use, snack intake, and lack of physical activity, as well as the motivation to resist these behavioral temptations. Third, we operationalized interpersonal communication as the frequency of discussions about the intervention (intervention condition only) and the three health behaviors outside class, and how positive or negative adolescents talk about the intervention and these three behaviors. The conversational frequency and valence are separately asked for conversations with friends, classmates, parents, and the teacher who taught InCharge. Fourth, regarding the program evaluation, we assess student perceptions of both the teacher and the prevention program InCharge. The included teacher-related measure is teacher communication, which is operationalized as a selection of variables assessing teacher clarity, verbal immediacy, and content relevance [36-38]. Evaluations of the program consist of an affective (eg, fun) and cognitive component (eg, informative).

#### Observational Data

Observations are conducted during delivery of the first and fourth lessons of InCharge to provide information on the classroom processes and how the program is delivered (Multimedia Appendices 2 and 3). The main outcomes of the observations are treatment integrity (ie, adherence to the protocol), interpersonal communication during the intervention, classroom interactional processes, teaching style, and student participation. First, treatment integrity or adherence is operationalized as the extent to which the delivered intervention resembles the intended intervention [39]. Additionally, we assess whether the teacher appears to support the intervention. Second, interpersonal communication is operationalized as the number of times a student or teacher talks about the three outcome behaviors during plenary discussions. We also assess whether a student or teacher initiates these discussions about the three outcome behaviors, whether comments during the plenary discussions are based on own experiences, and whether a student or teacher gives suggestions to increase self-efficacy regarding the three behaviors. Third, classroom interactional processes consist of three dimensions, namely emotional support (eg, positive climate), classroom organization (eg, behavior management), and instructional support (eg, quality of feedback [40]). Fourth, teaching style consists of the two components warmth and control [41]. Finally, we code several characteristics referring to student behavior, such as student participations, disruptive behaviors, and general class atmosphere.

#### Qualitative Data

The topic list of the focus groups with students includes interpersonal communication about temptations, evaluations of the InCharge program, and evaluations of the teacher. For interpersonal communication, we investigate the motivation to discuss temptations with the teacher, friends, classmates, and parents, and reasons why students did not discuss temptations with these conversational partners. The content as well as the valence of the conversations about temptations is investigated for interpersonal communication both within class and outside class. For the evaluation of the program, we aim to understand what parts of the intervention students find helpful or not, whether students actively complete in-class and homework assignments, and whether students think the intervention helps them to deal with temptations in the future. Lastly, we ask students about whether their teacher clearly explains the assignments during the intervention, who should ideally be given these interventions, and their perceptions of their teacher’s support for the intervention.

The topic list of interviews with teachers includes interpersonal communication, teacher perception of students, and evaluation of InCharge. For interpersonal communication, we assess the content and valence of conversations with students about the InCharge program, both during the intervention and outside class, as well as conversations that teachers have overheard among students. For teacher perception of students, we investigate how teachers perceive students’ participation and enjoyment. Finally, we discuss the health intervention with the teacher in order to find improvements for the program.

### Statistical Analysis

The background variables of the students (eg, gender, age, and educational level) will be checked to assess whether these variables are successfully randomized across the experimental and control conditions. Unsuccessfully randomized variables will be included as covariates in further analyses to control for potential confounding. Our data have a multilevel structure because participants are nested within classes, and these classes are nested within schools. Therefore, participants in one class or school are more similar than participants in other classes or schools. To account for this potential dependency in the data, we will correct standard errors and parameter estimates for clustering.

In order to determine the effectiveness of the health intervention, an intention-to-treat procedure and completers-only framework will be used to analyze the data. An intention-to-treat analysis of the data implies that participants will be analyzed under the condition that they are initially assigned, whereas completers-only framework analyzes only participants who are present at all time points. Using multilevel analyses, the three behavioral outcomes (snack intake, physical activity, and alcohol use) will be compared between participants in the experimental condition and those in the control condition at posttest and follow-up to determine the effects of the program. In the experimental condition only, multilevel analyses will be conducted to investigate the influence of treatment integrity on the predictors of the three health behaviors at posttest and follow-up. For missing data in multilevel models, maximum likelihood estimation will be used. As self-regulation is an important concept in InCharge, we will test whether the potential effects of InCharge on the health behaviors are explained by self-control. Additionally, relations between interpersonal communication with teachers, friends, classmates, and parents (ie, frequency and valence) and health-related outcomes, such as a mediating role between the conditions and outcomes, will be tested. As the program is delivered by teachers, we will also test in the experimental condition whether teacher communication relates to the evaluations of the health intervention and the three behavioral outcomes, using structural equation modeling. For structural equation models, expectation maximization will be used to impute the data.

For the qualitative data, interviews with teachers and focus groups with students will be analyzed on the strengths and weaknesses of the program in order to adapt the program. Furthermore, qualitative data will be used to determine the content of interpersonal communication with regard to the InCharge program and various temptations.

### Patient and Public Involvement

No patient is involved in this study.

### Ethics and Dissemination

The trial has been approved by the Ethics Review Board of the Faculty of Social and Behavioral Sciences of the University of Amsterdam (reference no.: 2017-PC-8244), and the trial is registered with the Netherlands Trial Register (NL6654). Manuscripts reporting the effectiveness of the health intervention and our other aims will be submitted to peer-reviewed journals for publication.

## Results

The recruitment, inclusion, and randomization of participants (ie, schools) started in the spring of 2017 and was continued in the spring of 2018, and data are being collected during the following two consecutive school years: 2017-2018 and 2018-2019. As of June 2019, a total of 1216 participants have been enrolled for this trial. This study is part of a PhD project and is expected to be completed in November 2020.

## Discussion

### Goals of the Study

The goal of this paper is to describe the study protocol of a cluster RCT that aims to evaluate the effectiveness of a school-based health intervention named InCharge. The goal of this program is to stimulate healthier lifestyles by improving the self-regulation skills of older adolescents. We expect that adolescents will be more inclined to refrain from binge drinking and eating unhealthy foods and to be more physically active after following the InCharge program compared with adolescents in the control condition.

### Strengths and Limitations

Given that InCharge is a school-based health intervention, the potential to reach a large number of adolescents is an important strength of the program. Second, the underlying mechanisms of InCharge are based on several often successfully tested and relevant theories such as the SCT [19] and the TPB [20], indicating that the program is theoretically well-founded. In addition to the theoretical foundation of the program, the content of InCharge is tailored to the developmental phase of older adolescents by including self-regulation as a core mechanism, which has been proven as one of the most effective prevention strategies during this developmental phase [12]. Furthermore, the study uses a mixed-method design with both quantitative and qualitative data to evaluate the effectiveness of the program. Another strength of the design is that it includes measures for immediate outcomes and measures for follow-up at 3 months, enabling us to assess both the short-term and medium-term effects of the prevention program in comparison with a control group. Lastly, research has shown that interpersonal communication about health topics strongly influences health behavior [16,17]. In our design, we investigate similar purposes of interpersonal communication, as well as the role of teacher communication, in the context of a school-based health intervention.

Our study has some limitations as well. The unit of randomization was classes instead of schools. The presence of both experimental classes and control classes within the same school could result in contamination between conditions because students from the experimental and control condition could have shared information about the intervention. However, as classes within the same school are generally more similar than classes from different schools, we expect that randomization at the class level will result in more comparable research conditions than randomization at the school level. Another limitation relates to the fact that the questionnaire data are obtained through self-reports. A disadvantage of self-reported data is that participants likely want to present themselves favorably, and hence, this might influence how they respond to surveys [42]. One example of such a bias in self-reports is the social desirability bias [43], which means that participants respond in a way that they believe would be perceived as socially desirable by others. This bias could, for example, lead participants to wrongly estimate the frequency of their health behaviors. To limit the incentive for social desirability, we assure participants about complete confidentiality and anonymity of their data.

### Practical Relevance

The InCharge study evaluates the effectiveness of the program, and its findings will help to further improve the program. The study also investigates whether including self-regulation as a key component in a school-based health intervention promotes healthier lifestyles among older adolescents. If findings reveal that improving self-regulation skills decreases unhealthy behaviors, such as alcohol use and snack intake, and increases healthy behaviors, such as physical exercise, the findings may aid the developers of health interventions in designing effective behavior change programs for older adolescents. In addition to the content of the intervention, investigating how teacher communication relates to the three health behaviors can provide information on how to communicate for a school-based health intervention. This information can be used to formulate communication guidelines in order to improve the delivery of school-based health interventions and, ultimately, healthier lifestyles. Promoting healthier lifestyles is especially important for older adolescents because unhealthy behaviors generally peak during late adolescence [4,5]. Finally, this study aims to provide information on how interpersonal communication during the intervention and outside the classroom could improve or hamper the effectiveness of school-based health interventions. These findings may help health care professionals in designing interventions that elicit desired conversations about health-related topics in order to stimulate healthier lifestyles.

### Conclusion

This protocol study uses an RCT to assess the effectiveness of InCharge, a newly developed school-based health intervention for older adolescents. As previous research has shown that utilizing self-regulation is an important prevention strategy, the goal of InCharge is to promote healthier behaviors, such as physical activity, and discourage unhealthy behaviors, such as alcohol use, by enhancing self-regulation skills. The program will be improved based on the findings of the effectiveness study.

## Appendix

Multimedia Appendix 1 Description of the assignments of the InCharge program.

Multimedia Appendix 2 Observation form of lesson 1.

Multimedia Appendix 3 Observation form of lesson 4.

## Abbreviations

RCT: randomized controlled trial
SCT: social cognitive theory
TPB: theory of planned behavior
