# Supplementary material for: Effectiveness of the InCharge Prevention Program to Promote Healthier Lifestyles: Protocol for a Randomized Controlled Trial
Source: JMIR Res Protoc. 2020 Jul 8;9(7):e17702. doi: 10.2196/17702 (PMC7381060; doi:10.2196/17702)
Supplement: Multimedia Appendix 1 [file resprot_v9i7e17702_app1.docx]

Appendix 1

Description of Assignments of the InCharge Program

**Lesson 1: My Temptations**

At the start of the lesson, students receive a worksheet containing the following assignments.

**Assignment 1: What do I want?**

In the first assignment, students are asked to think about where they want to be within five years, and how to get there. Together with their neighbor, students conduct interviews about their future goals and switch roles on the instructions of the teachers. Students are given several optional questions such as “What do you want to achieve with your education within the next five years?”, “What goals are most important to you and why?”, and “How would you want your relationship to be with the people most important to you after five years?” After the interviews are finished, the worksheet contains a statement “After five years, I want:” where students summarize their responses.

**Assignment 2: Temptations.**

The second assignment is a plenary one, in which classrooms are cleared and divided by an imaginary line. Students are asked to position themselves alongside this imaginary line, based on how tempting they assess certain temptations, which are chocolate or sweets, alcohol, staying in bed in the morning, skipping exercise, and procrastination of homework. After each statement, teachers ask students why they positioned themselves on either side of the imaginary line and stimulate discussions between students from opposite sides. After the statements, the classroom is reordered in its original state and students are asked to write down their personal temptations and why these temptations are so difficult to resist.

**Assignment 3: Your temptation & the future.**

The third assignment is an individual assignment, in which students are asked to imagine what would happen if they gave in to their temptation on a daily base and what would happen if they successfully resist their temptation for the next five years. The following questions are asked for giving in to and resisting their temptation: “How would you feel?”, “What would you look like?”, “How would this affect your education or job?”, “How would this affect your relationships with friends and family?”, “How satisfied would you be with yourself?”, and “What would others think about you?”. If students finish early, it is possible to answer these questions for a second temptation or describe more elaborately about the consequences of their temptation.

**Lesson 2: Willpower**

Students receive a worksheet containing the following assignments:

**Assignment 1: The importance of willpower.**

The first assignment is a plenary assignment, in which students watch a video of the Marshmallow experiment to understand the role of self-regulation. In this experiment young children are offered a choice between one marshmallow, provided immediately, or two marshmallows after waiting for a short period of time. Teachers ask questions about the video such as “What happened in the video?”, “How did the children resist the temptation, what techniques did they use?”, and “Fourteen years later, the researchers again studied these children who were then adults. How did the children that resisted the temptation differ from the children that did not resist the temptation?” Teachers inform students that children that resisted the temptation were now more successful and had fewer health problems compared to the children that did not resist the temptation. Additionally, students learn that willpower can be trained.

**Assignment 2: Your willpower!**

For the second assignment, teachers create groups of three students and let them discuss personal examples of challenges in which they successfully utilized willpower to achieve a certain goal. Suggested questions are “What was your goal or challenge?”, “How did you achieve this goal?”, “How did you feel when achieved this goal?”, and “How did your friends and family react to your success?” After the interviews are finished, teachers ask students to share some of their achievements with all other students and teachers ask students to write down their own responses on their worksheet.

**Assignment 3: 7-days challenge.**

In the third assignment, students resist a self-chosen temptation for seven days in order to train their willpower. Students have the option to do the entire challenge on their worksheet or a specific app for their phones. First, students formulate a goal that follows the SMART guidelines (specific, measurable, achievable, realistic, and timely). Second, students formulate an action plan for the challenge, in which students describe the benefits from succeeding in the challenge and think of a reward for if they succeed in the challenge. Additionally, students formulate how their challenge can be incorporated in their daily routine. For example, no more snacks after 09 PM. Finally, challenges may sometimes be more difficult and therefore, students think about a plan to overcome these situations using if-then statements. For example, if I want a snack after 09PM, then I take a glass of water instead. For each day in the challenge, students receive push notifications on their app to rate their day and describe the positive and negative aspects of that day in order to track their progress in the challenge. The app also contains advice to succeed in the challenge, such as “Communicate the challenge with friends and family, they can help you”, “Having a difficult time? Look for distraction such as texting a friend”, and “Stress affects your willpower, make sure you relax. Take a bath or a walk” For those that give in to their temptations, advice was given such as “Do not focus too much on your misstep. Think of the reasons why you do this challenge.”, “You have lost only if you stop trying.”, and “Don’t be too hard on yourself. It can happen to everyone. Stop thinking of it and continue with the challenge.”

**Lesson 3: Persist**

Students receive a worksheet containing the following assignments:

**Assignment 1: Experiences with the challenge.**

In the first assignment, a plenary discussion is held about the experiences of students with the 7-days challenge. Teachers ask questions such as “What did you think about the challenge?”, “What have you learned during the challenge?”, “What was difficult in doing the challenge?”, and “For those that succeeded, what would you advise the others?” After the plenary discussion about the student experiences, students write down the things that helped their challenge or made it more difficult.

**Assignment 2: Revise action plan.**

In the second assignment, students work in groups on the action plan of one of the group members that failed to do their challenge. First, the SMART guidelines are used to improve the goal of the challenge. For example, if the student experience that the goal is difficult to track during the challenge, students improve the goal by making it measurable. Second, students discuss the obstacles that the student experience during the challenge, and formulate if-then statements to overcome these obstacles. Finally, all students write down whether they would change anything if they could do the challenge again, and how they would do things differently.

**Assignment 3: Peer pressure.**

In the third lesson, students learn about the role of peer pressure because peer pressure is often a factor for deviation from action plans. To illustrate the effect of peer pressure, students watch a video of an experiment showing a participant sitting in a room together with confederates. As the room fills up with smoke, all confederates disregard the smoke and act like nothing happens. As a result, the participant conforms to the confederates and remains seated, illustrating conformity can be dangerous. After watching the video, teachers discuss the video with students and ask them questions such as “Why do the participants act the way they did?”, “What was the influence of the group?”, and “What role has peer pressure in resisting temptations?”

**Lesson 4: Alcohol**

In the fourth lesson, students receive a worksheet containing the following assignments:

**Assignment 1: Tough or a bungler?**

Students learn that peer pressure often has a role in alcohol use among adolescents. In the first assignment, students watch a video about adolescents on a holiday in Greece. The video shows a group of friends who are drinking. At first, they are having fun but later on conflict arises between the friends with an injury as result of excessive alcohol use. After the video, there is a plenary discussion in which students answer questions such as “What happened in the video?”, “What is your opinion of it?”, and “What role does peer pressure have in this situations?” For this assignment, teachers are instructed to discourage alcohol use and note the importance of delaying alcohol use.

**Assignment 2: Alcohol quiz.**

In the second assignment, students participate in a Kahoot quiz (digital quiz) using their phones about alcohol use. The goal of the quiz is to correct potential overestimations of alcohol use by portraying the actual drinking norms for same-aged peers. The quiz contains seven questions which are “How many percent of sixteen year olds have consumed alcohol in the past month?”, “About the video, do you think this is normal behaviour?”, “If a friend would behave like this, what would you think of it?”, “ How many percent of the sixteen-year old boys have been drunk in the past month?”, “How many sixteen-year olds have consumed more than 10 glasses of alcohol in the weekend?”, “How many young people were hospitalized because of alcohol abuse?”, and “Who believe that it is not a problem if their friends dot not drink?” Each question has two answer categories, and for the questions about student opinions, student receive no score. After each statement, teachers discuss the questions with the students.

**Assignment 3: Responsible use of alcohol.**

In assignment 3, students formulate action plans for alcohol use in pairs. First, there is a short plenary discussion about situations in which it is especially important to handle alcohol responsibly (e.g., during an exam week). Then, students choose an important situation themselves and formulate an action plan for alcohol use in pairs. Students think about certain temptations which may lead to deviation from their plans, and think of solutions to stick to their plans. Additionally, students formulate how they would respond to their friends if they invited them to drink. After formulating the plans, advices are shared in a plenary discussion to deal with peer pressure.
