# Supplementary material for: Effectiveness of the InCharge Prevention Program to Promote Healthier Lifestyles: Protocol for a Randomized Controlled Trial
Source: JMIR Res Protoc. 2020 Jul 8;9(7):e17702. doi: 10.2196/17702 (PMC7381060; doi:10.2196/17702)
Supplement: Multimedia Appendix 2 [file resprot_v9i7e17702_app2.docx]

Appendix 2

Observation Form of Lesson 1

**OBSERVATION FORM LESSON 1**

**School: ______________ Class: ______________ Number of students: ____ Date: ___________**

| **Introduction** | |
| --- | --- |
| 1. Where does the observer sit in the classroom? (from the perspective of the teacher) | |
| 1. What time does the lesson start? | |
| 3. How is the topic ‘temptations’ introduced? *(1 response possible)* □ Teacher reads the introduction out loud.  □ Students read the introduction individually.  □ Teacher introduces lesson by heart.  □ Teacher skips the introduction. | |
| 4. What elements are included in the introduction? (*more responses possible*)  □ People are exposed to temptations all day long  □ What is tempting, is different for everyone  □ Give one example of a temptation  □ Temptations can hinder personal goals  □ Temptations can have consequences  Did the teacher add extra information to the introduction? □ yes □ no If so, explain: | |
| 5. Most students were serious during the instruction | Not at all □□□□□ totally |
| Comments: | |

| **Block 1: What do I want?** | |
| --- | --- |
| 6. What time does the first assignment start? | |
| 7. How does the teacher introduce the first assignment? *(1 response possible)* □ Teacher reads the introduction out loud.  □ Students read the introduction individually.  □ Teacher introduces lesson by heart.  □ Teacher skips the introduction. | |
| 8. Does the teacher adhere to the protocol:   - The teacher divides the class in groups of two for an interview. □ ja □ nee - The teacher reminds students to reverse roles. □ ja □ nee - The teacher tells students to write a summary of their interview on the worksheets. □ ja □ nee - The teacher discusses the assignment with the students after they finished the assignment. □ ja □ nee - Did the teacher add extra information to the assignment? □ ja □ nee   If yes, explain: | |
| 9. Most students were serious during the first assignment | Not at all □□□□□ totally |
| 10. There is deviancy training during plenary discussions (exchanging of cool stories) □ yes □ no □ not able to observe   - - If so, the teacher responds adequately (cut it off, challenge to think differently, NOT: ignore or laugh). □ yes □ no | |
| 11. What time does the assignment end? | |
| Comments: | |

| **Block 2: Temptations** | |
| --- | --- |
| 12. What time does the second assignment start? | |
| 13. How does the teacher introduce the second assignment? *(1 response possible)* □ Teacher reads the introduction out loud.  □ Students read the introduction individually.  □ Teacher introduces lesson by heart.  □ Teacher skips the introduction. | |
| 14. How can students respond to the statements?  □ The classroom is separated in two halves which represent agree and disagree.  □ Students can position themselves alongside an imaginary line in the classroom varying from totally disagree to totally agree.  □ Students raise their hands if they agree.  □ Different, namely: | |
| \| 15. Check the statements that are discussed. Check a D if the teacher asks questions about the statement, and check an S if students talk to each other about the statement. \| Position of students towards the challenge: \| \| --- \| --- \| \| □ Homework D □ S □ \| Totally disagree □□□□□ Totally agree \| \| □ Sports D □ S □ \| Totally disagree □□□□□ Totally agree \| \| □ Sleep D □ S □ \| Totally disagree □□□□□ Totally agree \| \| □ Snacks D □ S □ \| Totally disagree □□□□□ Totally agree \| \| □ Alcohol D □ S □ \| Totally disagree □□□□□ Totally agree \| | |
| 16. Does the teacher adhere to the protocol:   - Teacher shows and reads the PowerPoint presentation. □ yes □ no - Teacher discusses the statements shortly with the students. □ yes □ no - Teacher asks students about their points of view. □ yes □ no - Teachers stimulate students with different perspectives to provide arguments for their statement. □ yes □ no - Teacher asks students to write down their biggest temptation. □ yes □ no - Did the teacher add extra information to the assignment? □ yes □ no   If so, explain: | |
| 17. Most students were serious during the second assignment. | Not at all □□□□□ totally |
| 18. There is deviancy training during plenary discussions (exchanging of cool stories) □ yes □ no □ not able to observe  If so, the teacher responds adequately (cut it off, challenge to think differently, NOT: ignore or laugh). □ yes □ no | |
| 19. What time does the assignment end? | |
| Comments: | |

| **Blok 3: Your temptation and the future** | |
| --- | --- |
| 20. What time does the third assignment start? | |
| 13. How does the teacher introduce the third assignment? *(1 response possible)* □ Teacher reads the introduction out loud.  □ Students read the introduction individually.  □ Teacher introduces lesson by heart.  □ Teacher skips the introduction. | |
| 22. Does the teacher adhere to the protocol:   - Teacher asks students to describe the consequences of giving in to their temptation every day for the next five years. □ yes □ no - Teacher asks students to describe the consequences of resisting their temptation every day for the next five years. □ yes □ no - Teacher discusses the assignment in a plenary discussion after students are finished. □ yes □ no - Did the teacher add extra information to the assignment? □ yes □ no   If so, explain: | |
| 23. Most students were serious during the third assignment. | Not at all □□□□□ totally |
| 24. There is deviancy training during plenary discussions (exchanging of cool stories) □ yes □ no □ not able to observe  If so, the teacher responds adequately (cut it off, challenge to think differently, NOT: ignore or laugh). □ yes □ no | |
| 25. What time does the assignment end? | |
| 26. Extra: Did the teacher give the homework assignment? □ yes □ no | |
| Comments: | |

FILL OUT AFTER LESSON FINISHED (SAME FOR LESSON 1 AND LESSON 4)

| **Alcohol, physical inactivity and snacks** |
| --- |
| 27. Who initiated these topics?   - Alcohol: □ N/A □ Teacher and students □ teacher □ students - Insufficient exercise: □ N/A □ Teacher and students □ teacher □ students - Snacks: □ N/A □ Teacher and students □ teacher □ students |
| 28. How often did the teacher talk about the following topics during the lesson?   - Alcohol: □ Not at all □ once □ twice □ three times □ more often - Insufficient exercise: □ not at all □ once □ twice □ three times □ more often - Snacks: □ not at all □ once □ twice □ three times □ more often |
| 29. Does the teacher talk more positively or negatively about these topics?   - Alcohol: Only negative □□□□□ Only positive - Insufficient exercise: Only negative □□□□□ Only positive - snacks: Only negative □□□□□ Only positive |
| 30. Did the teacher talk about his/her own experience with these topics?  Were these experiences positive or negative?   - Alcohol □ yes □no Only negative □□□□□ Only positive - Insufficient exercise: □ yes □no Only negative □□□□□ Only positive - Snacks: □ yes □no Only negative □□□□□ Only positive |
| 31. Does the teacher talk about their self-efficacy regarding these three behaviors? (e.g., tips to drink less alcohol?   - Alcohol: □ yes □ no - Insufficient exercise: □ yes □ no - Snacks: □ yes □ no explain: |
| 32. How often did the students talk about the following topics during the lesson?   - Alcohol: □ not at all □ once □ twice □ three times □ more often - Insufficient exercise □ not at all □ once □ twice □ three times □ more often - Snacks: □ not at all □ once □ twice □ three times □ more often |
| 33. Do the students talk more positively or negatively about these topics?   - Alcohol: Only negative □□□□□ Only positive - Insufficient exercise: Only negative □□□□□ Only positive - Snacks: Only negative □□□□□ Only positive |
| 34. Did the students talk about their own experiences with these topics? Were these experiences positive or negative?   - Alcohol: □ yes □no Only negative □□□□□ Only positive - Insufficient exercise: □ yes □no Only negative □□□□□ Only positive - Snacks: □ yes □no Only negative □□□□□ Only positive |
| 35. Did the students talk about their self-efficacy regarding these three behaviors? (e.g., tips to drink less alcohol?   - Alcohol: □ yes□ no - Insufficient exercise: □ yes□ no - Snacks: □ yes□ no Explain: |

| **Teacher** |
| --- |
| 36.  **Emotional support:**  To what extent does the teacher contribute to a positive classroom climate? (*e.g., shows affection and respect to the students*)  not at all □□□□□ totally To what extent does the teacher contribute to a negative classroom climate? *(e.g., shows aggression and lack of respect to the students*)  not at all □□□□□ totally To what extent does the teacher focus on the needs of the students? (e.g., *teacher walks through classroom to offer support where needed)*  not at all □□□□□ totally To what extent does the teacher match the motivation, interests, and visions of the students? (*e.g., allows students to talk during plenary discussions)*  not at all □□□□□ totally Explain:  **Organization of the classroom:**  To what extent does the teacher make clear how students should behave, and present specific rules so that little time is wasted to misbehavior of students?  not at all □□□□□ totally To what extent is the teacher efficient during the instructions, so that students have sufficient time to learn from the lessons? (through assignments and discussions)  not at all □□□□□ totally To what extent does the teacher involve students in the lesson?  not at all □□□□□ totally Explain:  **Instructional support:**  To what extent does the teacher stimulate students to think for themselves, rather than simply transferring information?  not at all □□□□□ totally To what extent is the feedback of the teacher aimed at learning and understanding, rather than what is right or wrong? (*e.g., teacher asks for arguments*)  not at all □□□□□ totally To what extent does the teacher stimulate interactions between students?  not at all □□□□□ totally Explain: |
| Comments about the teacher: |

| **Teacher** |
| --- |
| 37. *loyalty to the intervention:*  To what extent does the teacher adhere to the protocol of InCharge? not at all □□□□□ totally  To what extent does the teacher seem to support the intervention? not at all □□□□□ totally  To what extent does the teacher communicate with the observer? not at all □□□□□ totally |
| 38. *Teaching style:*  Teacher shows interest in students. not at all □□□□□ totally  Teacher shows that he/she cares about the students. not at all □□□□□ totally  Teacher has routines/rules for how students should behave not at all □□□□□ totally  during plenary discussions.  Teacher keeps an eye on the student’s behavior. not at all □□□□□ totally |
