# Supplementary material for: Effectiveness of the InCharge Prevention Program to Promote Healthier Lifestyles: Protocol for a Randomized Controlled Trial
Source: JMIR Res Protoc. 2020 Jul 8;9(7):e17702. doi: 10.2196/17702 (PMC7381060; doi:10.2196/17702)
Supplement: Multimedia Appendix 3 [file resprot_v9i7e17702_app3.docx]

Appendix 3

Observation Form of Lesson 4

**OBSERVATION FORM LESSON 4**

**School: ______________ Class: ______________ Number of students: ____ Date: ___________**

| Introduction | |
| --- | --- |
| 1. Where does the observer sit in the classroom? (from the perspective of the teacher) | |
| 1. What time does the lesson start? | |
| 3. How is the topic ‘alcohol as a temptation’ introduced? *(1 response possible)* □ Teacher reads the introduction out loud.  □ Students read the introduction individually.  □ Teacher introduces lesson by heart.  □ Teacher skips the introduction. | |
| 1. What elements are included in the introduction? (*more responses possible*)   □ Temptations can interfere with personal goals  □ Willpower can be a strong influence.  □ Alcohol is a well-known temptation.  □ Refusing alcohol can be difficult among friends.  □ In this lesson, the principles of previous lessons are applied to alcohol.  Did the teacher add extra information to the introduction? □ yes □ no If so, explain: | |
| 1. Most students were serious during the instruction | Not at all □□□□□ totally |
| Comments: | |

| Block 1: Tough or a bungler? | |
| --- | --- |
| 1. What time does the first assignment start? | |
| 1. How does the teacher introduce the first assignment?   □ Teacher reads the introduction out loud.  □ Students read the introduction individually.  □ Teacher introduces lesson by heart.  □ Teacher skips the introduction. . | |
| 1. Does the teacher adhere to the protocol:  - Teacher shows video fragment. □ yes □ no - Teacher discusses video fragment with the students in a plenary discussion. □ yes □ no - Teacher stimulates students to postpone alcohol use after 18 years. □ yes □ no - Teacher makes it clear that regular alcohol use is not normal for young people. □ yes □ no - Teacher discusses the assignment in a plenary discussion. □ yes □ no - Did the teacher add extra information to the assignment? □ yes □ no   If so, explain: | |
| 1. Most students were serious during the first assignment | Not at all □□□□□ totally |
| 1. There is deviancy training during plenary discussions (exchanging of cool stories) □ yes □ no □ not able to observe  - If so, the teacher responds adequately (cut it off, challenge to think differently, NOT: ignore or laugh). □ yes □ no | |
| 1. What time does the assignment end? | |
| Comments: | |

| Block 2: The alcohol quiz | |
| --- | --- |
| 1. What time does the second assignment start? | |
| 1. How does the teacher introduce the assignment? (*1 response possible*)   □ Teacher reads the introduction out loud.  □ Students read the introduction individually.  □ Teacher introduces lesson by heart.  □ Teacher skips the introduction. | |
| 1. How was the quiz conducted?   □ Via the app.  □ Via the PowerPoint.  □ Teacher reads the statements. | |
| 1. Check the questions that are being discussed. Check a D when the teacher asks students about the statements, and check an S if students talk to each other about the statements.   . | Comments: |
| □ Percentage that have not drunk. □ D □ S |  |
| □ Excessive alcohol use. □ D □ S |  |
| □ Opinion about drunken friend. □ D □ S |  |
| □ Percentage drunk boys. □ D □ S |  |
| □ 10 glasses of alcohol. □ D □ S |  |
| □ Hospitalization. □ D □ S |  |
| □ No problem if friends consume no alcohol. □ D □ S |  |
| 1. Does the teacher adhere to the protocol.  - Teacher discusses the statements in a plenary discussion after each statement. □ yes □ no - Teacher leads the discussion. □ yes □ no - Teacher makes it clear that excessive alcohol use is not normal. □ yes □ no - Teacher discusses the assignment after the quiz is finished. □ yes □ no - Did the teacher add extra information to the assignment? □ yes □ no   If so, explain: | |
| 1. Most students were serious during the second assignment. Not at all □□□□□ totally | |
| 1. There is deviancy training during plenary discussions (exchanging of cool stories) □ yes □ no □ not able to observe   If so, the teacher responds adequately (cut it off, challenge to think differently, NOT: ignore or laugh). □ yes □ no | |
| 1. What time does the assignment end? | |
| Comments: | |

| Blok 3: Responsible use of alcohol |
| --- |
| 1. What time does the third assignment start? |
| 1. How does the teacher introduce the third assignment? (*1 response possible*)   □ Teacher reads the introduction out loud.  □ Students read the introduction individually.  □ Teacher introduces lesson by heart.  □ Teacher skips the introduction. |
| 1. Does the teacher adhere to the protocol?  - Teacher asks examples of situations in which alcohol should be consumed responsibly □ yes □ no - Teacher divides the students in groups of two. □ yes □ no - Teacher discusses the action plans in a plenary discussion. □ yes □ no - Did the teacher add extra information to the assignment? □ yes □ no   If yes, explain: |
| 1. Most students were serious during the third assignment. Not at all □□□□□ totally |
| 1. There is deviancy training during plenary discussions (exchanging of cool stories) □ yes □ no □ not able to observe  - If so, the teacher responds adequately (cut it off, challenge to think differently, NOT: ignore or laugh). □ yes □ no |
| 1. What time does the assignment end? |
| 1. Extra: Did the teacher give the homework assignment? □ yes □ no |
| Comments: |
